# In *Helicobacter pylori *auto-inducer-2, but not LuxS/MccAB catalysed reverse transsulphuration, regulates motility through modulation of flagellar gene transcription

**DOI:** 10.1186/1471-2180-10-210

**Published:** 2010-08-06

**Authors:** Feifei Shen, Laura Hobley, Neil Doherty, John T Loh, Timothy L Cover, R Elizabeth Sockett, Kim R Hardie, John C Atherton

**Affiliations:** 1 Centre for Biomolecular Sciences, University of Nottingham, University Park, Nottingham NG7 2RD, UK; 2 Nottingham Digestive Diseases Centre NIHR Biomedical Research Unit, School of Clinical Sciences, University of Nottingham and Nottingham University Hospitals NHS Trust, Nottingham NG7 2UH, UK; 3 Institute of Genetics, School of Biology, Queen's Medical Centre, University of Nottingham NG7 2UH, UK; 4 Department of Medicine, Vanderbilt University School of Medicine, Nashville, TN 037232-2605 and Veterans Affairs Tennessee Valley Healthcare System, Nashville, TN 37212, USA; 5 School of Molecular Medical Sciences, University of Nottingham, University Park, Nottingham NG7 2RD, UK; 6 Current Address: Department of Veterinary Medicine, University of Cambridge, Cambridge CB3 0ES, UK; 7 Current Address: Department of Food Sciences, Sutton Bonington Campus, University of Nottingham, Leicestershire LE12 5RD, UK

## Abstract

**Background:**

LuxS may function as a metabolic enzyme or as the synthase of a quorum sensing signalling molecule, auto-inducer-2 (AI-2); hence, the mechanism underlying phenotypic changes upon *luxS *inactivation is not always clear. In *Helicobacter pylori*, we have recently shown that, rather than functioning in recycling methionine as in most bacteria, LuxS (along with newly-characterised MccA and MccB), synthesises cysteine via reverse transsulphuration. In this study, we investigated whether and how LuxS controls motility of *H. pylori*, specifically if it has its effects via *luxS*-required cysteine metabolism or via AI-2 synthesis only.

**Results:**

We report that disruption of *luxS *renders *H. pylori *non-motile in soft agar and by microscopy, whereas disruption of *mccA*_Hp _or *mccB*_Hp _(other genes in the cysteine provision pathway) does not, implying that the lost phenotype is not due to disrupted cysteine provision. The motility defect of the Δ*luxS*_Hp _mutant was complemented genetically by *luxS*_Hp _and also by addition of *in vitro *synthesised AI-2 or 4, 5-dihydroxy-2, 3-pentanedione (DPD, the precursor of AI-2). In contrast, exogenously added cysteine could not restore motility to the Δ*luxS*_Hp _mutant, confirming that AI-2 synthesis, but not the metabolic effect of LuxS was important. Microscopy showed reduced number and length of flagella in the Δ*luxS*_Hp _mutant. Immunoblotting identified decreased levels of FlaA and FlgE but not FlaB in the Δ*luxS*_Hp _mutant, and RT-PCR showed that the expression of *flaA, flgE*, *motA*, *motB*, *flhA *and *fliI *but not *flaB *was reduced. Addition of DPD but not cysteine to the Δ*luxS*_Hp _mutant restored flagellar gene transcription, and the number and length of flagella.

**Conclusions:**

Our data show that as well as being a metabolic enzyme, *H. pylori *LuxS has an alternative role in regulation of motility by modulating flagellar transcripts and flagellar biosynthesis through production of the signalling molecule AI-2.

## Background

Many bacteria release extra-cellular signalling molecules (auto-inducers) to perform intercellular communication. It is generally assumed that auto-inducers are employed to regulate aspects of bacterial behaviour in response to cell population density (so-called quorum sensing). This includes changes in the expression of genes crucial for bacterial survival or virulence [1,2]. Auto-inducer-2 (AI-2) production is widespread among bacterial species; its formation is catalysed by the enzyme LuxS [3]. Many Gram-positive and Gram-negative bacterial species possess LuxS, and in some it has been shown to catalyse AI-2 production and to control quorum sensing (QS). Good examples include *Vibrio harveyi *and *Vibrio cholera*, where AI-2 has been shown to regulate density-dependent bioluminescence and virulence factor production, respectively [4,5]. *luxS *inactivation has also been shown to cause phenotypic alterations such as biofilm formation, changes in motility, toxin production, and reduced colonisation in various experimental infection models [3,6].

In addition to its QS role, LuxS catalyses one of the steps of the activated methyl cycle (AMC). The AMC is a central metabolic pathway that generates the *S*-adenosylmethionine (*S*AM) required by methyltransferases allowing the widespread methylation of proteins and DNA needed for cell function. It recycles the toxic product of these reactions, *S*-adenosylhomocysteine (*S*AH), to help provide the cell with sulphur-containing amino acids [7]. As part of the AMC, the Pfs enzyme, 5'-methylthioadenosine nucleosidase/S-adenosylhomocysteine nucleosidase converts *S*AH to *S*-ribosylhomocysteine (*S*RH) which is subsequently converted to homocysteine by LuxS. The precursor of AI-2, 4, 5-dihydroxy-2, 3-pentanedione (DPD) is generated as a by-product of this reaction. Through a process of dehydration and spontaneous cyclisation, some or all of the DPD is rearranged into a cocktail of chemically related molecules known as AI-2, including 4-hydroxy-5-methyl-3 (*2H*) furanone, (*2R*, *4S*) -2-methyl-2, 3, 3, 4-tetrahydroxy-tetrahydrofuran and furanosyl borate diester. These have been shown to function as signals of communication between bacteria [3,8,9]. In some organisms, the AMC is different. For example, in *Pseudomonas aeruginosa*, LuxS and Pfs are replaced by a single enzyme (*S*AH hydrolase) which converts *S*AH to homocysteine in a one step reaction without the concomitant production of DPD [7].

*Helicobacter pylori*, a Gram-negative bacterium which causes peptic ulceration, gastric cancer and gastric mucosa-associated lymphoid tissue (MALT) lymphoma, contains a *luxS *homologue and produces AI-2 [10-12]. *luxS*_Hp _(HP0105_26695_; JHP0097_J99_) is positioned next to housekeeping genes *mccA*_Hp _(HP0107_26695_; JHP0099_J99_) and *mccB*_Hp _(HP0106_26695_; JHP0098_J99_) on the *H. pylori *chromosome, in a putative operon [13-15]. Data from our laboratory have demonstrated that the AMC of *H. pylori *is incomplete, and that LuxS_Hp_, MccA_Hp _and MccB_Hp _constitute the sole cysteine biosynthetic pathway in this bacterium via a reverse transsulphuration pathway (RTSP) [15].

To date, the mechanisms underlying phenotypic changes exhibited as a result of *luxS*_Hp _inactivation remain elusive. Two Δ*luxS*_Hp _mutants have been shown to form biofilms more efficiently than the parent strain, indicating a possible but counterintuitive role of *luxS*_Hp _in biofilm reduction [16]. A subsequent study demonstrated that Δ*luxS*_Hp _mutants in two strains lost growth-phase-dependent regulation of the gene encoding the major flagellin FlaA, and that cell culture supernatant containing AI-2 could increase *flaA *transcription [17]. Studies by two independent groups looked at fitness of Δ*luxS*_Hp _mutants *in vivo *using mouse and gerbil models, respectively [18,19]. The motility of Δ*luxS*_Hp _mutants was diminished and bacterial fitness reduced in co-infection experiments. Restoration of *luxS*_Hp _by genetic complementation partially restored these phenotypes [18,19]. The authors argued that the decreased fitness in the Δ*luxS*_Hp _mutant was most likely due to the disruption of the cycle of *S*RH consumption and homocysteine synthesis and that AI-2 seemed unlikely to be a QS signal molecule [18]. More recently however, Rader *et al. *reported that *luxS*_Hp _disruption affected flagellar morphology in the absence of one of the transcriptional regulators (σ^28^, *flgS *or *flgM*), and that this could be complemented upon the addition of DPD. They reported that loss of *luxS*_Hp _caused decreased transcription of the flagellar regulator *flhA*, and that expression of *flhA *was induced by DPD [20]. This complementation through the addition of exogenous DPD resurrected the possibility of LuxS-dependent signalling in *H. pylori*.

There are several possible mechanisms whereby a motility defect could be associated with loss of *luxS*_Hp_. Firstly, reduced flagellar structural gene transcription and related protein synthesis would lead to loss of flagella. Secondly, normal flagella structures may be synthesised in the Δ*luxS *mutant but lack of a functional motor may prevent rotation. Thirdly, both motor and flagellum may be functional, but unable to respond to tactic signals, leading to aimless movement.

In this study, we set out to distinguish between the mechanisms underlying the alteration in motility of Δ*luxS*_Hp _mutants, and to clarify whether this originated from a disruption of metabolism or QS. To do this, electron microscopy was employed to examine flagellar assembly and the levels of individual components of flagella were assessed at a transcriptional and translational level. Our demonstration here of the lack of motility defects in mutants disrupted in components of the RTSP other than LuxS, coupled to the inability of cysteine to complement the motility defect of the Δ*luxS*_Hp _mutant, shows that disruption of cysteine biosynthesis is not the mechanism underlying the reduction in motility. In contrast, we show that exogenously added AI-2 (or DPD) influences motility via regulating flagellar gene transcription (and thus the number and length of flagella). This supports the existence of an additional role for LuxS in *H. pylori *as a signalling molecule synthase.

## Methods

### Strains and growth culture conditions

All strains used in this study are listed in Table 1. DH5α was used in the production of proteins needed for AI-2 biosynthesis and cloning [21]. *V. harveyi *BB170 was used in the bioluminescence bioassay as a reporter strain [22]. *E. coli *strains were routinely grown in Luria-Bertani (LB) (Bacto) broth or on agar plates at 37°C. *V. harveyi *was grown in LB or AB medium [23] at 30°C, also under normal atmospheric conditions. *H. pylori *strains were routinely grown and maintained on Columbia blood agar plates (No.2, with 5% [v/v] horse blood; Oxoid) or grown in Brucella broth (BB) (Bacto) containing 7% (v/v) fetal bovine serum (Gibco). *H. pylori *J99 was incubated at 37 °C for 24 h to 72 h as required in a MG500 VAIN-cabinet (Don Whitley Scientific) in an atmosphere of 5% CO_2_, 86% N_2_, and 6% O_2 _(all v/v). For motility experiments the method of Wand *et al. *[24] was used to achieve motile cultures for analysis, see below. Antibiotics were used at the following concentrations: ampicillin at 100 μg/ml, kanamycin at 30 μg/ml.

**Table 1:** Strains and plasmids used in this study

Strains/Plasmids	Description	Reference
Strains		
*Vibrio harveyi*		
BB170	*luxN *:: Tn5 AI-1 sensor negative; AI-2 sensor positive	[43]
		
*Escherichia coli*		
DH5α	*endA*1 *recA*1 *gyrA96 thi-1 hsdR17*(r_k_^- ^m_k_^+^) *relA1 supE44Δ(lacZYA-argF) U169 F^- ^Φ80dlacZΔM15 deoA phoA λ^-^*	[21]
DH5α LuxS	DH5α containing the plasmid pProEx-*luxS*_EC_	[8]
DH5α Pfs	DH5α containing the plasmid pProEx HT mtan	[8]
		
*Helicobacter pylori*		
J99 (ATCC700824)	Wild-type motile strain	[44]
J99 Δ*luxS*	J99 derivative; Δ*luxS *:: *km*; Km^r^	[15]
J99Δ*luxS*-F	J99 derivative; Δ*luxS *:: *km*-*sacB*; Km^r ^Suc^s^	This study
J99 Δ*luxS*^+^	J99Δ*luxS*-F derivative; Δ*luxS *:: *km*-*sacB *replaced with original *luxS *locus; Suc^r ^Km^s^	This study
J99 Δ*mccA*	J99 derivative; Δ*mccA *:: *km*; Km^r^	[15]
J99 Δ*mccB*	J99 derivative; Δ*mccB *:: *km*; Km^r^	[15]
J99 Δ*flhB*	J99 derivative; ΔHP0770 Lys^13 ^to Glu^347^; Km^r^; non-motile	[24]
CCUG 17874*	Wild-type strain	[29]
17874 Δ*flaA*	17874 derivative; Δ*flaA *:: *cat*; Cm^r^	Paul O'Toole
17874 Δ*flgE*	17874 derivative; Δ*flgE *:: *km*; Km^r^	[30]
		
Plasmids		
pGEMT	Commercial TA cloning vector; Amp^r^	Promega
pGEMTluxSXN396	pGEM-T with inserted 26695 *luxS*; Δ*luxS *:: *km*-*sacB*; Suc^s ^Km^r^	[17]
pGEMTluxS	pGEM-T with inserted full-length *luxS *fragment	This study
pProEx-*luxS*_EC_	pProEX HT containing the *luxS *gene of *E. coli *MG1655	[8]
pProEx HT mtan	PProEX HT containing the *pfs *gene of *E. coli*	[8]

* CCUG 17874 is identical to the type strain NCTC 11637, isolated by B. J. Marshall at Royal Perth Hospital, May 1982 [29].

### Molecular biology methods

Preparation of plasmid DNA, DNA ligation, gel electrophoresis and transformation of *E. coli *strains were performed in accordance with standard methods [25]. All PCRs were performed with Taq DNA polymerase (Roche Diagnostics, Lewes, UK). TA cloning was carried out using the pGEM-T vector system (Promega, Madison, WI). Plasmid DNA was extracted using the QIAquick spin miniprep kit (QIAGEN, UK). DNA fragments were purified from agarose gel using a QIAquick gel extraction kit (QIAquick, UK) according to the manufacturer's instruction. *H. pylori *genomic DNA was isolated as described previously [26]. DNA sequencing was conducted using standard fluorescent dye terminator chemistries, and analysis performed using the Applied Biosystems 3730 DNA Analyzer system (Geneservice, Cambridge, UK, Applied Biosystems Inc, Foster City, CA.). Results were analysed using the Bioedit software suite [27].

### Construction of the complemented Δ*luxS^+ ^*strain

*H. pylori *J99 wild-type was transformed with the plasmid pGEMTluxSXN396 containing a *km-sacB *construct encoding kanamycin resistance (Km^r^) and (5%) sucrose sensitivity (Suc^s^) [17]. Disruption of the chromosomal *luxS *gene was accomplished by natural transformation, allelic exchange, and screening for kanamycin-resistance as previously described [15], resulting in the J99 Δ*luxS *mutant strain. The presence of the *km-sacB *cassette was verified by amplifying fragments of *H. pylori *chromosomal DNA using primers *luxS*-F/*luxS*-R (forward, 5'>GTG GCT TTA GCG GGA TGT TTT<3'; reverse, 5'>GCGA ACA AAT CCC CGC TG<3') and DNA sequencing. The J99 Δ*luxS *was then transformed with plasmid pGEMTluxS (encoding wild-type *luxS*), and transformants in which *km-sacB *had been replaced with the introduced original *luxS *locus were selected for sucrose resistance on medium containing 5% sucrose and screened for kanamycin sensitivity. The presence of the original *luxS *gene was verified by amplifying fragments on *H. pylori *chromosomal DNA using primers *luxS*-F/*luxS*-R and DNA sequencing.

### Bacterial growth curves and *V. harveyi *bioluminescence assay

Bacterial broth cultures were started from a blood agar plate culture, diluted to an OD_600 nm _of 0.05 in fresh BB medium, and grown at 37°C in a VAIN-cabinet with shaking. OD_600 nm _measurements were taken at the 6 h, 24 h, 48 h and 72 h time points, and at the same time cell suspensions were harvested and filtered through a 0.2 μm pore size filter. The AI-2 activity in cell free supernatants (CFS) was tested as previously described using the *V. harveyi *reporter strain BB170 [9,22]. Briefly, an overnight *V. harveyi *culture was diluted 1:2500 in fresh AB medium [23]. CFS samples were diluted 1:10 in the AB medium containing BB170 into the 96 well bioluminescence plates to give a final volume of 200 μl and were incubated at 30°C. The bioluminescence and optical density were determined at 30 min intervals for at least 8 h using a luminometer (Anthos Labtech LUCY 1.0). AI-2 activity alterations in bioluminescence were expressed as induction (n-fold) over the negative control.

### Motility assay

Plate motility assay of *H. pylori *was performed in Brucella broth medium (BD Biosciences), supplemented with 7% (v/v) fetal bovine serum (Gibco), 0.35%-0.45% (w/v) agar (No.1, Oxoid) and the indicator, 40 μg/ml triphenyl tetrazolium chloride (Sigma, UK). Inclusion of this indicator made it easier to see the small recombinant colonies. Plates were seeded with 5 μl *H. pylori *liquid culture (forming a circle with 3 mm diameter) standardised to an OD_600 nm _of 1.0 and were incubated at 37°C for up to 7 days under the conditions described above. The motility halos were recorded using a digital camera and the area of each halo was measured using a GS-800 Calibrated Densitometer (Biorad).

Motility analysis was also carried out by direct observation under phase-contrast microscopy using a Nikon Eclipse E600 after cells were grown in co-culture conditions as used by Wand *et al. *[24]. Briefly, co-cultures of *H. pylori*-human gastric adenocarcinoma (AGS) cells were prepared (described below). After 24 h, 10 μl culture was placed onto a microscope slide and covered with a coverslip and freely-motile *H. pylori *cells were analysed under the microscope.

### Plate motility bioassay using chemically defined media (CDM)

The liquid chemically defined media were prepared as previously described [15,28]. 60 ml of sterile chemically defined media were added to 40 ml of molten 1% Oxoid No. 1 agar base to make 0.4% semi-solid chemically defined agar. Cysteine supplemented plates (CSP) were made by adding cysteine to the molten agar, shortly before it set. The final concentration of cysteine was 1.0 mM, which was non-limiting for *H. pylori *growth. The centre of each plate was seeded with one-day incubated *H. pylori *cells and was incubated for 5 days under the conditions described above. The motility halos were recorded using a digital camera and the area of each halo was measured using a GS-800 Calibrated Densitometer (Biorad).

### Motility assay with AI-2 complementation

AI-2 was synthesised enzymatically as described previously using purified proteins LuxS*_E. coli _*and Pfs*_E. coli _*[8]. For complementation of the Δ*luxS*_Hp _motility phenotype, soft motility agar plates (0.4% w/v) were made as previously described. Bioluminescence activity of the AI-2 product was quantified using the *V. harveyi *bioassay and compared to CFS from *H. pylori *wild-type broth culture standardised to an OD_600 nm _of 1.0 at the time point in the growth curve that maximal AI-2 activity was measured. 1/400 diluted *in vitro *synthesised AI-2 product shows the same level of bioluminescence as seen in the *H. pylori *wild-type CFS in the *V. harveyi *bioassay. Therefore, in the complementation experiment AI-2 was added to motility plates to a final concentration of 0.25% (v/v). 24 h *H. pylori *cultures were seeded individually onto the centre of each motility plate and incubated for 5 days. The area of outward migration was recorded with a digital camera and measured using a GS-800 Calibrated Densitometer (Biorad).

### Tissue culture and bacterial co-culture

All chemicals were obtained from Gibco, UK. AGS cells were grown in nutrient mixture Ham's F-12 supplemented with L-glutamine (200 mM) and fetal bovine serum (Gibco) (10% v/v) in a 37°C incubator containing 5% CO_2_. After cells had grown to confluency, a 1 in 5 or 1 in 8 dilution was added to a 75 cm^2 ^flask containing fresh media mix and incubated in the same conditions as before to allow cells to re-grow to confluency.

AGS cells were counted using the trypan (0.35% v/v) blue dye method. Cells were seeded at a density of 1 × 10^5 ^cells/ml into 6 well plates and grown to 80% confluency. The cell-media mix was removed and replaced with 2 ml fresh F-12 media. Plates were inoculated with 24 h *H. pylori *liquid cultures standardised to an OD_600 nm _of 0.1 and incubated for one day in a microaerobic environment. Bacterial cells were then analysed using a phase-contrast Nikon Eclipse E600 microscope and electron microscopy.

### Electron microscopy (EM)

*H. pylori *cells were pre-grown as described above for motility analysis. 15 μl of culture was allowed to settle on a carbon formvar grid (Agar Scientific) for 1 min. The suspension was removed and the grid washed by addition of 15 μl of Phosphate Buffered Saline (PBS) for an additional minute. This was removed and the cells stained with 0.5% Phospho-tungstic acid (PTA) pH 7.0 for 1 min. Grids were examined and pictures taken using a JEOL JEM1010 Transmission Electron Microscope. We quantified changes, rounding to the nearest 5% and quote means ± SD. Essentially, three groups of *H. pylori *cell samples prepared on different dates were examined. Each group of samples contained wild-type, Δ*luxS *and Δ*luxS*^+ ^cells treated and not treated with DPD. For each group, 100 *H. pylori *cells from each culture sample were examined.

### Cysteine and DPD complementation experiments

Cysteine from Sigma products was dissolved in distilled water according to the manufacturer's recommendation. Synthetic DPD was purchased from Omm Scientific Inc. DPD (AI-2) activity was quantified with the bioluminescence bioassay and compared to wild-type *H. pylori *grown to an OD_600 nm _of 1.0, at which maximal AI-2 activity was obtained. To test for complementation of motility, DPD (at a physiological concentration of 150 μM) and non-limiting cysteine (1.0 mM) were added individually to bacteria-AGS cell co-cultures. DPD was added after 10 h of incubation and once again after 18 h of incubation. Cysteine was added from the beginning of incubation. Bacterial motility and cells were observed and visualized by phase-contrast microscope and EM, respectively. For gene transcription studies, DPD (150 μM) and cysteine (1.0 mM) were also added (in the same way) individually to *H. pylori *liquid cultures of different genotypes. After 24 h, RNA was extracted and the transcript levels of genes of interest were measured.

### Protein electrophoresis and western blotting

*H. pylori *wild-type, its Δ*luxS*_Hp _mutant, the complemented Δ*luxS*_Hp_^+ ^mutant and controls (*H. pylori *wild-type 17874 [29], and derived mutants Δ*flaA *(a kind gift from Paul O'Toole) and Δ*flgE *[30]) were grown in Brucella broth at 37°C for up to 24 h, at which point high levels of AI-2 activity were detected. To exclude global differences in protein production between strains, we corrected our loading for numbers of bacteria rather than for total protein levels. To do this, 24 h liquid (Brucella broth) culture of each strain was adjusted to OD_600 nm _of 1.0. A 500 μl cell sample of each strain was then centrifuged at 5500 rpm for 1 min. Culture supernatants were removed and cell pellets were fully resuspended in 1 ml sterile PBS. 100 μl protein sample was collected. The same volume of 2 × sample buffer was added and boiled for 10 min. SDS-polyacrylamide gel electrophoresis (SDS-PAGE) and subsequent immunoblotting were carried out as described previously under standard conditions [25]. The gel contained 10% acrylamide. 4 μl protein stock from each strain sample was loaded into each well of the SDS-PAGE gel. For immunoblotting, proteins were transferred from SDS-PAGE gels to nitrocellulose paper by the methanol Tris-glycine system described by Towbin *et al. *[31]. To see whether similar amounts of protein were loaded using our methodology, membranes were inspected following Ponceau red staining prior to immunoblotting; protein levels appeared similar on each membrane by inspection. The blots were incubated with rabbit polyclonal antibodies against *H. pylori *flagellin and hook protein (a generous gift from Paul O'Toole) [32]. Bound antibodies were detected using secondary anti-rabbit IgG alkaline phosphatase conjugate antibody (Sigma, UK). The blots were developed using the BCIP/NBT substrate system (Dako, UK). The quantitative scan of the protein bands was performed using a GS-800 Calibrated Densitometer (Biorad). The reflective density (RD) of each protein band was measured using the Quantity One 4.6.5 software (Biorad).

### RNA extraction and transcription analysis

RNA was isolated from *H. pylori *cells grown in BB medium for 24 h. Cultures were treated with RNA protection reagent (QIAGEN, UK) and RNA was extracted using RNeasy mini kit (QIAGEN, UK). Contaminating genomic DNA was removed using a DNA free kit (Ambion). Synthesis of cDNA was performed using Ominiscript RT kit (QIAGEN, UK) and random hexamers (Roche, Germany). Quantitation of transcripts of selected genes of interest was accomplished by quantitative reverse transcription-PCRs (qRT-PCRs) using Rotor-gene 3000. Primers utilised in RT-PCRs are listed in Table 2. All RT-PCR reaction mixtures contained 12.5 μl of SYBR Green Mix (QIAGEN, UK), 5 μl of gene specific primers, 2 μl cDNA template (cDNA was diluted 10-fold prior to adding into the RT-PCR reactions) and RNase free water to a final volume of 25 μl. The amplification program was 95°C for 15 min, followed by 35 cycles of 95°C for 15 sec, 56°C for 60 sec, and 72°C for 30 sec. All samples, including the controls (*16 S *rRNA and no-template), were run in triplicate. Transcript levels of each gene were normalised to the *16 S *rRNA in each sample. The relative quantity of transcription of each gene was obtained using Pfaffl's analytical methodology.

**Table 2:** Primers utilised in quantitative RT-PCR

Primers	Sequence (5'-3')
16S_F	CGA TGA AGC TTC TAG CTT GC
16S_R	ATA GGA CAT AGG CTG ATC TC
flaAF	CAG GTT CGT ATC GCT ACA GGC
flaAR	ATC ACT TCT GCT AAC ACG CCG
flaBF	ACT GGG ATT GGG GCG TTA
flaBR	TCA ACC TCC CGTCAG CGT C
flgEF	GCT CAG GCA CGA TCA CTC TAA
flgER	AAC GCC ATG AAA GGT CTT AAT AC
flhAF	TCA TTG GAG GGT TTT TAG TGG
flhAR	GGT GCG AGT GGC GAC AAT
motAF	TGA GTT TAG AGG GGC GAG TG
motAR	CCA GTA ATG AGC GGC ACC
motBF	TTC AGG GAA AGA AGA AGA GCA A
motBR	TCA AAC AGC AAA CTA GAG AAA A
fliIF	ACG AGC GAT GAT AGC CCT TTA
fliIR	ACC GAT TTC TCT TTG AGC CAT
ureAF	GAT GAT GTG ATG GAT GGT GTG G
ureAR	TAA GAA CAA CTC ACC AGG AAC C

### Statistics

Statistical analysis was by Student's *t *test.

## Results

### The *H. pylori *Δ*luxS *mutant lost the ability to produce AI-2 while the wild-type, Δ*mccA*_Hp _and Δ*mccB*_Hp _mutants did not

**Figure 1:**
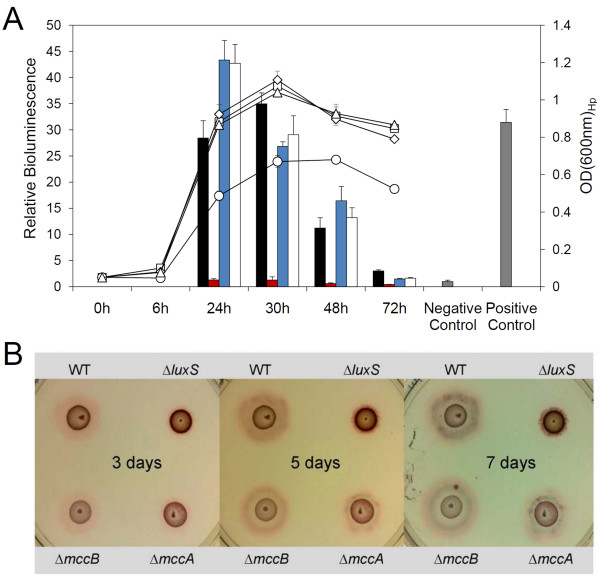
**The Δ*luxS*_Hp _mutant of *H. pylori *strain J99 lacks AI-2 and is non-motile unlike other mutants deficient in cysteine biosynthesis**. (A) AI-2 production in J99 wild-type (black column), Δ*luxS*_Hp _(red column), Δ*mccB*_Hp _(blue column) and Δ*mccA*_Hp _(white column) mutants was measured. Strains were grown in Brucella broth, and aliquots were removed at 24 h, 30 h, 48 h and 72 h to assess the optical density (wild-type, square; Δ*luxS*_Hp_, diamond; Δ*mccB*_Hp_, circle; Δ*mccA*_Hp_, triangle) and the amount of AI-2 in the filtered culture supernatant using the *V. harveyi *bioassay. AI-2 activity is shown as a relative bioluminescence (corrected by OD_600nm _of *H. pylori*) in the presence of *H. pylori *culture supernatants over the negative control (Brucella broth alone). A diluted *in vitro *synthesised AI-2 sample was utilised as a qualitative positive control [8]. Bioluminescence induced by wild-type, Δ*mccB*_Hp_, and Δ*mccA*_Hp _strains was significantly greater than that induced by the Δ*luxS*_Hp _mutant, as determined by paired Student's *t*-test (*p *< 0.001). The lines represent the growth (OD, righthand axis) and the bars represent the AI-2 activity (bioluminescence, lefthand axis). (B) 5 μl of liquid culture (24 h) of the wild-type, Δ*luxS*_Hp_, Δ*mccB*_Hp _and Δ*mccA*_Hp _mutants was seeded on each quarter of a soft agar plate. After 3, 5 and 7 days of incubation, the motility halo of each strain was recorded using a digital camera. All experiments were done in triplicate: a representative experiment is shown and the mean results are presented in the text.

Our previous study has demonstrated that *luxS*_Hp_, *mccA*_Hp _and *mccB*_Hp _genes comprise a reverse transulphuration pathway in *H. pylori*, which is the sole cysteine biosynthesis pathway [15]. We then wanted to determine whether these mutants in a motile strain of *H. pylori*, J99, would be useful in differentiating whether *H. pylori *motility was affected by *luxS *associated AI-2 production or by cysteine provision. Firstly, we needed to establish whether mutations in *mcc*_Hp _genes in our candidate motile strain J99 changed expression of *luxS*_Hp _and AI-2 biosynthesis. To do this, *H. pylori *J99 wild-type and derived Δ*mccA*_Hp_, Δ*mccB*_Hp_, and Δ*luxS*_Hp _mutants were grown in Brucella broth containing serum (10% v/v). Once they reached logarithmic growth phase, AI-2 activity in the culture supernatant was measured using the *V. harveyi *AI-2 bioassay previously described [4,8]. As expected, the wild-type produced AI-2 in a growth dependent manner, with AI-2 accumulating during the late logarithmic phase, and reaching maximal levels in the stationary phase. During stationary phase, AI-2 levels decreased and were almost undetectable by 72 h. Similar data were obtained with Δ*mccA*_Hp _and Δ*mccB*_Hp _mutants, despite the fact that the Δ*mccB*_Hp _mutant grew slightly less well than the other mutants and the wild-type. The Δ*luxS*_Hp _mutant, unlike the wild-type and the other two mutants, yielded almost undetectable levels of bioluminescence at each time point, indicating that the production of AI-2 is *luxS*_Hp_-dependent and that insertion of a kanamycin cassette (*aphA3*) into *mccA*_Hp _and *mccB*_Hp _did not affect expression of the downstream gene *luxS*_Hp _(Figure. 1A).

### Deletion of *luxS*_Hp _abolishes motility while the Δ*mccA*_Hp _and Δ*mccB*_Hp _mutants remained motile

To investigate whether motility of *H. pylori *was affected by cysteine biosynthesis, we first compared the motility of *H. pylori *wild-type with Δ*luxS*_Hp_, Δ*mccA*_Hp _and Δ*mccB*_Hp _mutants. To do this, a 24 h liquid culture of each strain was spotted onto each quarter of a semi-solid agar plate and incubated for up to 7 days. The resulting motility halo areas were quantified after 3, 5 and 7 days of incubation. Halo areas that surrounded the wild-type, Δ*mccA*_Hp _and Δ*mccB*_Hp _strains kept increasing during continuous incubation, although the Δ*mccA*_Hp _strain was slightly delayed in comparison to the others. After 7 days of culture, the Δ*luxS*_Hp _mutant remained almost non-motile and produced a significantly (*p *< 0.001) reduced motility halo compared to wild-type, Δ*mccA*_Hp _and Δ*mccB*_Hp _strains in 3 independent repeat experiments (Figure. 1B). After 7 days, the wild-type, Δ*mccA*_Hp _and Δ*mccB*_Hp _mutants produced halos of (mean ± SD) 8.5 ± 0.6 mm, *n *= 4; 5.6 ± 0.9 mm, *n *= 4; and 7.8 ± 0.6 mm, *n *= 4 increases in diameter, respectively, all significantly greater than the Δ*luxS*_Hp _mutant which produced a halo size of 1.1 ± 0.1 mm, *n *= 4. These results revealed that the reduction in motility was likely a result peculiar to *luxS*_Hp _mutation rather than due to disruption of cysteine biosynthesis.

### Genetic complementation or exogenous AI-2 can restore the motility defect of the Δ*luxS*_Hp _mutant, but exogenous cysteine addition cannot

**Figure 2:**
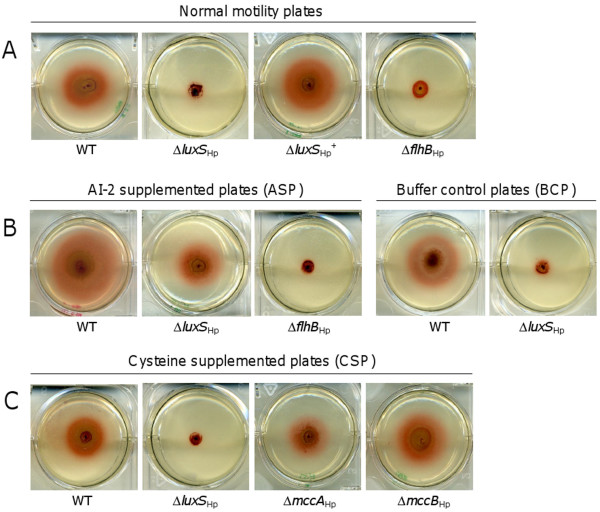
**AI-2, but not cysteine rescues the motility defect of the ΔLuxS_Hp _mutant**. (A) Wild-type, Δ*luxS*_Hp_, and Δ*luxS*_Hp_^+ ^bacteria were seeded onto soft plates composed of normal medium. The non-motile Δ*flhB *mutant served as the negative control. (B) Wild-type, Δ*luxS*_Hp _and Δ*flhB*_Hp _bacteria were seeded onto motility plates supplemented with *in vitro *synthesised AI-2. Wild-type and Δ*luxS*_Hp _were also seeded on motility plates containing buffer control solution used for *in vitro *AI-2 synthesis. (C) Wild-type, Δ*luxS*_Hp _Δ*mccA*_Hp _and Δ*mccB*_Hp _strains were seeded onto chemically defined motility plates supplemented with cysteine. After 5 days of incubation, the motility halo of each strain on each plate was recorded using a digital camera and the area of each halo was measured using a GS-800 Calibrated Densitometer (Biorad).

To rule out the possibility that second site mutations in the Δ*luxS*_Hp _mutant were inhibiting motility, genetic complementation was performed to create the Δ*luxS*_Hp_^+ ^strain (see Materials and Methods). The non-motile Δ*flhB *mutant was used as a negative control [24]. 24 h cultures of wild-type, Δ*luxS*_Hp_, Δ*luxS*_Hp_^+ ^and Δ*flhB*_Hp _strains grown in Brucella broth were individually spotted onto normal motility plates. After 5 days of incubation, the mean halo diameter of the Δ*luxS*_Hp_^+ ^strain was 6.9 ± 0.2 mm, *n *= 4, which was slightly larger than that of the wild-type (4.7 ± 0.7 mm, *n *= 4). The Δ*luxS*_Hp _and Δ*flhB*_Hp _mutants showed non-motile phenotypes (Figure. 2A).

To examine whether AI-2 can influence the motility of *H. pylori*, we assessed the motility of the wild-type, Δ*luxS*_Hp _and Δ*flhB *mutants on AI-2 supplemented plates (ASP). The ASP was prepared using 0.4% soft agar containing *in vitro *synthesised AI-2 (0.25% v/v). The buffer control plate (BCP) was also produced using 0.4% soft agar into which was added the buffer control solution (0.25% v/v) produced in parallel to *in vitro *AI-2 synthesis (buffer containing no AI-2). After 5 days of incubation, the halo size of the wild-type on ASP increased by 11.2 ± 0.7 mm, *n *= 4, compared with a 5.4 ± 0.2 mm, *n *= 4 increase on the non-supplemented plate (compare Figure. 2A or the right panel of Figure. 2B with the left panel of Figure. 2B). Whilst the Δ*luxS*_Hp _mutant was non-motile on the BCP, the halo increased by 4.6 ± 0.4 mm, *n *= 4 on ASP (Figure 2B). The control strain Δ*flhB*_Hp _mutant remained non-motile on the ASP (Figure. 2B).

Having established an influence on motility for one of the chemicals reliant on LuxS_Hp _function (AI-2), we sought to establish whether another (cysteine) would have a similar influence. Our previous studies revealed that exogenous cysteine rescues growth defects of mutants unable to complete cysteine biosynthesis via the RTSP of *H. pylori *(Δ*luxS*_Hp_, Δ*mccA*_Hp _and Δ*mccB*_Hp _mutants) in chemically defined broth [15]. Chemical complementation of motility was thus performed using chemically defined plates supplemented with 1.0 mM cysteine. Methionine was added to these plates as the sulphur source since all known *H. pylori *strains are methionine auxotrophs. After 5 days of incubation, wild-type *H. pylori *and Δ*mccA*_Hp _and Δ*mccB*_Hp _mutants formed motility halos of 4.9 ± 0.3 mm, *n *= 4; 3.6 ± 0.6 mm, *n *= 4; and 4.3 ± 0.9 mm, n = 4 increases in diameter, respectively. The Δ*luxS*_Hp _mutant remained non-motile (Figure. 2C).

Taken together, these data indicate that the motility defect of the Δ*luxS*_Hp _mutant was restored either genetically or chemically with AI-2, but not with exogenous cysteine. This suggests that *luxS *and AI-2 play a role in enhancing bacterial motility, rather than an intact cysteine biosynthesis pathway, implying a likely role of *luxS*_Hp _in signalling.

### ΔLuxS_Hp _mutants have altered flagella morphology and motility patterns

Motility plates effectively indicate motility phenotypes of the population, but do not give any indication of the structure of the motility organelles (flagella), or the motility pattern of individual cells. To characterise the phenotypes underlying the decreased ability of the Δ*luxS*_Hp _mutant to swarm in soft agar, we examined motility of individual bacterial cells using phase-contrast microscopy and also the flagellar morphology of the cells using electron microscopy. Cells tested included wild-type, Δ*luxS*_Hp _and Δ*luxS*_Hp_^+^, all grown in the presence and absence of DPD and cysteine. All cells were grown in co-culture with human gastric adenocarcinoma (AGS) cells for 24 h before testing, as previous experiments in our laboratory have shown that this gives highly reproducible results in *H. pylori *motility experiments.

Phase-contrast microscopy revealed that > 40% of wild-type and Δ*luxS*_Hp_^+ ^cells were motile; whereas less than 2% of Δ*luxS*_Hp _cells were motile. When grown with exogenous DPD, motile cells again made up > 40% of the population for wild-type and Δ*luxS*_Hp_^+ ^cells, but now also made up > 40% of the population for Δ*luxS*_Hp _cells. Cultures of the Δ*luxS*_Hp _grown with exogenous cysteine consistently contained less than 2% motile cells. To exclude the possibility that the restoration of motility of Δ*luxS*_Hp _cells was due to an effect of DPD on AGS cells rather than on *H. pylori*, we set up a control sample in which the wild-type and Δ*luxS*_Hp _mutant were co-cultured individually with AGS cells that had been treated with DPD overnight. DPD was washed off with the media before co-culturing. As expected, both wild-type and Δ*luxS*_Hp _cells in these control cultures showed very similar motility phenotypes to those co-cultured with normal AGS cells, indicating that DPD is a functional signalling molecule to *H. pylori *cells rather than it working through affecting eukaryotic cells. Moreover, the approximate speed of motile Δ*luxS*_Hp _cells was visibly lower compared to the wild-type, Δ*luxS*^+ ^and all cell samples plus DPD.

**Figure 3:**
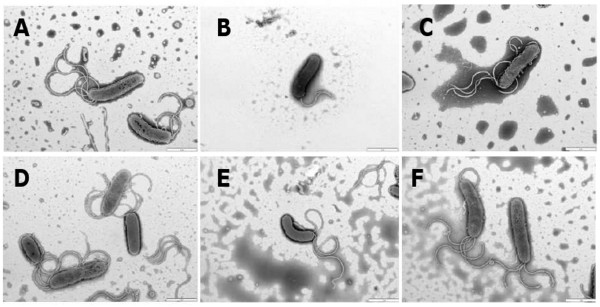
***luxS*_Hp_/DPD modulates flagellar morphogenesis**. *H. pylori *cells were co-cultured with AGS cells. Cells were stained with 0.5% photungstate (PTA). Scale bars represent 2 μm. (A) wild-type, (B) Δ*luxS*_Hp_, (C) Δ*luxS*_Hp_^+^, (D) wild-type with DPD, (E) Δ*luxS*_Hp _with DPD and (F) Δ*luxS*_Hp_^+ ^with DPD. DPD was added after 10 h of incubation and once again after 18 h of incubation during co-cultures.

Electron microscopic images (Figure. 3) showed that all samples tested (wild-type, Δ*luxS*_Hp _and Δ*luxS*_Hp_^+^, grown in the presence or absence of DPD) produced a flagellar filament of some kind in the majority of bacterial cells, but those of the Δ*luxS*_Hp _strain were consistently short and usually fewer in number. In our experiments, nearly all of the wild-type cells tested had flagella (95% ± 3%, n = 3) and most of these had multiple flagella, which were usually at one pole and typically 3-4 in number (90% ± 3%, n = 3) (Figure. 3A). In contrast, fewer Δ*luxS*_Hp _cells tested had flagella (70% ± 5%, n = 3) and these were typically shorter and also fewer in number (30% ± 5%, n = 3 of cells had only one or two short flagella (Figure. 3B)). The complemented Δ*luxS*_Hp_^+ ^cells were similar to wild-type, with nearly all cells possessing 3-4 normal long flagella at least one pole (95% ± 3%, n = 3) (Figure. 3C). Addition of DPD to Δ*luxS*_Hp _cells also converted them to a wild-type morphology, with the vast majority producing 3-4 wild-type length flagella usually present at a single pole (95% ± 3%, n = 3) (Figure. 3E). Addition of DPD to wild-type cells had little significant effect with nearly all remaining flagellate as before (95% ± 3%, n = 3) although more cells were seen with a flagellum at both poles (Figure. 3D). Addition of DPD to the Δ*luxS*_Hp_^+ ^cells had a similar effect, with more cells with flagella at both poles (Figure. 3F).

### Mutation of *luxS*_Hp _resulted in the decreased production of flagellar proteins FlaA and FlgE

**Figure 4:**
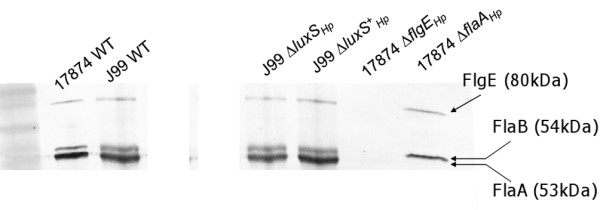
**Mutation of *luxS*_Hp _causes altered flagellin and hook protein production**. Cell lysates of the strains indicated were subjected to immunoblotting with anti-flagellin (FlaA and FlaB) and anti-hook protein (FlgE) together [32]. The proteins were measured in wild-type, Δ*luxS*_Hp_, Δ*luxS*_Hp_^+ ^cultures grown in Brucella broth at 37°C for 24 h. *H. pylori *strain 17874 wild-type [29] served as the positive control. Mutants in *flaA *(a kind gift from Paul O'Toole) and *flgE *[30] derived from this strain (17874Δ*flaA *and 17874Δ*flgE*) served as negative controls for identifying FlaA and FlgE, respectively.

The reduced number and length of flagella in Δ*luxS*_Hp _cells observed by electron microscopy could emanate from a number of different changes in the proteome. As previous work had suggested possible involvement of major flagella proteins, we investigated these first by immunoblotting whole cell lysates. Cell lysates were adjusted so that protein from equivalent numbers of bacteria was loaded (see Materials and Methods), and probed with anti-flagellin (FlaA and FlaB) and anti-FlgE (hook protein) antiserum (Figure. 4). In practice, FlaB levels were very similar between all wild-type and mutant strains and were not shown to vary in our subsequent transcription analysis. Our main aim here was to compare ratios of flagella proteins between wild-types and mutants, so we expressed results of other flagella proteins (FlaA and FlgE) relative to FlaB levels within each strain. *H. pylori *wild-type 17874, and derived mutants (Δ*flaA *and Δ*flgE*) were used as positive and negative controls, respectively. In our experiments, four repetitions were included, when the reflective density (RD) of each protein band was measured using Quantity One 4.6.5 software (Biorad).

We found that all strains tested produced FlaB at approximately the same level (Figure. 4). The reflective density of the FlaB bands of the wild-type, Δ*luxS*_Hp _mutant and the complemented Δ*luxS*_Hp_^+ ^mutant were (means ± SD) 0.210 ± 2.0E-03 RD, n = 4; 0.204 ± 5.8E-04 RD, n = 4; and 0.207 ± 5.8E-04 RD, n = 4, respectively. We expressed all other results (FlaA and FlgE) relative to FlaB in each strain. Mutagenesis of LuxS_Hp _reduced the expression of FlaA relative to FlaB (from mean 1.60 in the wild-type to 1.23 in the Δ*luxS*_Hp _mutant, *p *< 0.01), and complementation increased the ratio back to wild-type levels (mean 1.70 in the Δ*luxS*_Hp_^+ ^mutant, *p *< 0.01 compared with the Δ*luxS*_Hp _mutant). Next, we examined FlgE expression, and a similar trend was found (wild-type FlgE:FlaB ratio mean 0.74; Δ*luxS*_Hp _mutant 0.51; complemented Δ*luxS*_Hp_^+ ^mutant 0.77; *p *< 0.01 for differences between Δ*luxS*_Hp _mutant and wild-type and complemented stains). These data show that FlaA and FlgE synthesis was reduced relative to FlaB in the Δ*luxS*_Hp _mutant and these changes were restored by genetic complementation.

### AI-2 regulates the transcription of flagellar genes

**Figure 5:**
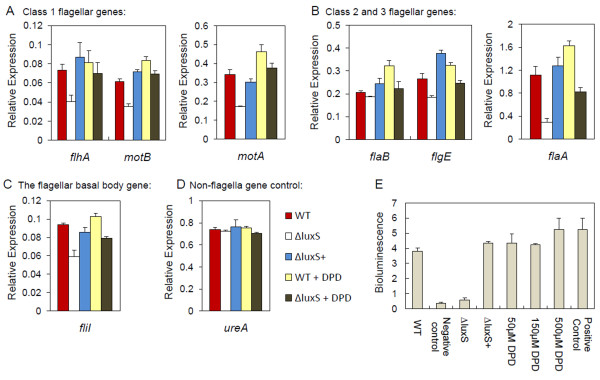
***luxS*_Hp_/DPD modulates *H. pylori *flagellar gene transcription**. Transcript levels of (A) *flhA*, *motA*, *motB*; (B) *flaB*, *flgE*, *flaA*; (C) *fliI *were determined by qRT-PCR normalised to the levels of the *16 S *rRNA gene. (D) Relative expression of *ureA *was utilised as a non-flagella gene control. The Y axis shows the relative transcriptional level of each gene in each strain normalised to the level of the same gene in the strain control (which is J99 wild-type in every case). Values are mean activities of triplicate RNA samples of each strain. Transcript levels were measured in wild-type and Δ*luxS*_Hp _cultures grown with or without DPD (150 μM) and in Δ*luxS*_Hp_^+ ^cultures grown without DPD. (E) AI-2 activity (using the previously-described *V. harveyi *BB170 bioluminescence assay [4]) in DPD solution (at concentrations of 50 μM, 150 μM or 500 μM) and in cell free culture supernatant (24 h) of *H. pylori *wild-type, Δ*luxS*_Hp _and Δ*luxS*_Hp_^+ ^strains grown in the Brucella broth (starting OD_600 nm _of 0.05). Negative control is Brucella broth alone. A diluted *in vitro *synthesised AI-2 sample was utilised as a qualitative positive control [8]. Error bars indicate standard deviation.

Previous reports have provided evidence that *luxS*_Hp_-dependent QS may occur to modulate motility via transcriptional regulation of *flaA *or *flhA *[20]. We utilised quantitative RT-PCR (qRT-PCR) to screen for alterations in transcription of these and other genes involved in flagellar assembly to extend our understanding of the regulatory mechanisms that might be involved. To exclude an effect of cysteine biosynthesis, exogenous addition of cysteine was also undertaken. The concentration of cysteine was non-limiting to *H. pylori *growth. *16 S *rRNA transcription was used for normalization and *ureA *served as a non-flagella linked gene control (Figure. 5D).

The flagellar genes tested included several from different regulatory hierarchy positions in flagellar synthesis [33]: class 1 genes *flhA *(encodes flagellar regulator component), *motA *and *motB *(encode flagellar motor proteins); class 2 genes *flaB *(encodes hook-proximal minor flagellin) and *flgE *(enodes flagellar hook protein); and class 3 gene *flaA *(encodes major flagellin). *fliI *(encodes membrane-associated export ATPase of the flagellar basal body) was also examined (Figure. 5).

For class 1 genes tested, *flhA *showed a consistent pattern of 1.75 fold reduced transcription (*p *< 0.001), and both *motA *and *motB *showed a consistent pattern of 2 fold (*p *< 0.001) reduced transcription in the Δ*luxS*_Hp _mutant compared to the wild-type (Figure. 5A). For class 2 genes tested, *flgE *was 1.5 fold (*p *< 0.001) down-regulated in the Δ*luxS*_Hp _mutant; while *flaB *did not exhibit any significant change. *flaA *was the only class 3 gene tested, which was 3.5 fold (*p *< 0.001) down-regulated in the Δ*luxS*_Hp _mutant compared to the wild-type (Figure. 5B). Additionally, the transcript of *fliI *was also significantly (1.5 fold, *p *< 0.001) decreased in the mutant (Figure. 5C).

The reduced transcription of *flhA*, *motA*, *motB*, *flgE*, *flaA *and *fliI *was restored genetically by the complementation of the mutant with the wild-type *luxS*_Hp _gene. Also, 150 μM DPD was sufficient to restore the transcription of these genes in the Δ*luxS*_Hp _mutant to levels similar to the wild-type (Figure. 5E). Although Figure 5E shows that 50 μM and 150 μM DPD induced almost the same level of bioluminescence as the wild-type, we chose to use 150 μM DPD in the complementation experiment because this concentration was shown to be more reproducible (it has the smaller error bar). In wild-type cells, addition of DPD markedly increased transcription of *motA*, *motB*, *flaA *and *flaB*, whilst *flhA*, *flgE *and *fliI *only showed a marginal increase. Exogenous addition of cysteine to the Δ*luxS*_Hp _mutant did not significantly increase transcription of any of the genes studied; suggesting that addition of cysteine was not able to restore the transcription of flagellar genes (data not shown). Consistent with the analysis of protein levels, these RT-PCR data indicate that *luxS*_Hp _disruption has a greater effect upon transcription of *flaA *than of *flaB*. Taken together, these data suggest that the effect of LuxS in cysteine metabolism does not regulate expression of flagellar genes, and that the effects on flagellar gene transcription are likely through AI-2 production.

## Discussion

The function of *luxS*_Hp _is controversial due to putative roles both in signalling and metabolism. Disruption of cysteine biosynthesis by independent mutations that had no influence on AI-2 production did not alter motility. In contrast, the motility defect of a Δ*luxS*_Hp _mutant of *H. pylori *was genetically complemented by *luxS*_Hp_, or chemically complemented by the addition of exogenous AI-2 but not by exogenous cysteine. The processes underlying the loss of motility of the Δ*luxS*_Hp _mutant were manifested by fewer and shorter flagella that presumably derived from the altered flagella protein production and the modulated expression of a number of genes linked with flagella assembly and function.

Previous studies have shown that mutations of *luxS*_Hp _in *H. pylori *diminished motility on soft agar. The altered motility phenotype was restored completely by genetic complementation with *luxS*_Hp _or significantly restored by metabolic complementation with wild-type CFS [18-20]. In contrast to our study, in Osaki *et al. *and Rader *et al*.'s studies complementation of *luxS*_Hp _was performed by placing *luxS*_Hp _at a second site in the chromosome rather than at the original locus [19,20]. Like these previous reports, our study shows that abolished motility of J99 Δ*luxS*_Hp _mutation was restored entirely by complementation with the *luxS*_Hp _gene and significantly by *in vitro *synthesised AI-2. The previous studies, with complete complementation of motility with *luxS*_Hp _through insertion at a new chromosomal locus, argue against polar effects of *luxS*_Hp _mutagenesis on other genes which influence motility. Our study, with complementation with *luxS*_Hp _through creating a revertant results in similar levels of LuxS_Hp _to wild-type and thus better shows that the phenotypes attributed to the mutant were not due to secondary mutations elsewhere in the chromosome.

Furthermore, having demonstrated that MccA_Hp _and MccB_Hp _function consecutively to convert the product of LuxS_Hp _(homocysteine) into cysteine as part of the RTSP [15], we reasoned that inactivation of any of these three enzymes would have a similar influence upon cysteine biosynthesis, whilst only the Δ*luxS*_Hp _mutant would be devoid of AI-2. Thus, if the reduced motility of the Δ*luxS*_Hp _mutant derived from disrupted cysteine biosynthesis, mutants in *mccA*_Hp _and *mccB*_Hp _would have a similar motility defect. Therefore, we performed an experiment to exclude the possibility that the effect on motility was due to non-specific secondary metabolic effects of LuxS_Hp_. To do this, wild-type, Δ*luxS*_Hp_, Δ*mccA*_Hp _and Δ*mccB*_Hp _strains were inoculated on the same motility plate, allowing the production of AI-2 and the biosynthesis of cysteine to be isolated from each other. As expected, only the Δ*luxS*_Hp _mutant was non-motile. This, for the first time, suggests that motility of *H. pylori *cannot be affected by disrupting the cysteine provision pathway, but can be blocked by the loss of *luxS*_Hp _itself. By using a chemically defined medium, we confirmed the provision of cysteine had no effect on motility of *H. pylori*.

Earlier publications have suggested that AI-2 may not act as a signal in some bacteria but instead may simply be a by-product of the important AMC pathway [9]. In support of this, in some bacteria, production of AI-2 does appear to be associated with metabolic rather than regulatory phenomena [34]. However, data from our motility bioassays using both motility plates and microscopy demonstrate that in *H. pylori *AI-2 (or DPD) controls motility. In our experiments, the shorter flagella observed in the mutant could result from the observed alteration in the FlaA:FlaB ratio as previously described [35,36]. However, proving this would require extensive immuno-EM analysis with anti-FlaA and anti-FlaB antisera, which is beyond the scope of this work. As *flaA *has been confirmed to be essential for motility in *H. pylori *while *flaB *is a structural subunit of the flagellar filament which increases motility [35,36], the change of the ratio between flagellins FlaA and FlaB may be one factor resulting in the abolished motility of the Δ*luxS*_Hp _mutant. Also, LuxS_Hp_/AI-2 appears to affect the position of flagella, suggesting that LuxS_Hp_/AI-2 may affect genes involved in the formation of flagella at the cell poles.

The reduced expression of flagellar motor genes (*motA *and *motB*) which control flagellar rotation may be a further factor contributing to slower motility of the Δ*luxS*_Hp _mutant although it could also be caused by the lower flagellar number requiring fewer motor units to encircle each flagellar base. Thus it is likely that the flagella in the Δ*luxS*_Hp _strain are too short and too few to form effective flagellar propellers to produce *Helicobacter *movement. This is in contrast to a previous report where truncated flagella were only reported in G27 strains that also lacked one of the transcriptional regulators (σ^28^, *flgS *or *flgM*) and where wild-type length flagella were reported for the Δ*luxS*_Hp _mutant alone [20]. However, surprisingly in that report, the addition of DPD to the double mutants lengthened the flagellar filaments.

Mutants defective in *flhA *were previously described as being defective in flagellar apparatus assembly and in motility. Recently Rust and coworkers (2009) reported that the anti-sigma factor for σ^28^, FlgM, interacts with FlhA at the base of the *Helicobacter *flagellum and this interaction modulates the expression of flagellar genes by σ^28 ^[37]. The decrease in *flhA *expression, seen in our Δ*luxS*_Hp _mutant could explain the change in flagellar length but not via a FlgM-dependent pathway as seen by Rader *et al. *[20], as Rust and coworkers report that FlgM levels were wild-type in a Δ*flhA *mutant in *Helicobacter *strains N6 and 88-3887 [37].

Both Rust and co-workers [37] and Neihus and co-workers [33] show that FlaB is not regulated by the same regulatory pathway as FlaA, and as FlaB levels in our Δ*luxS*_Hp _mutant concur with this, the short flagella we observe in the Δ*luxS*_Hp _mutant are likely to be predominantly composed of FlaB (normally hook-proximal) flagellins. These may be extended, to give functional length propellers by synthesis and assembly of FlaA in wild-type filaments and in filaments from *luxS*_Hp_*-*complemented Δ*luxS*_Hp_^+ ^bacteria or Δ*luxS*_Hp _bacteria+DPD which have longer flagella.

FlaB and FlgE are both part of the regulon that is controlled by the FlgS/FlgR two component system and the sigma factor σ^54 ^(RpoN) [33]. Interestingly, though no significant change in FlaB was found, FlgE production as well as its gene expression was affected by loss of LuxS/AI-2. This suggests that *luxS *inactivation might affect transcription of the same class of flagellar genes differently. One possibility is that the FlgR/FlgS-σ^54 ^regulatory complex might have different effects on the same class of genes when affected by loss of LuxS; another possibility is that there may be additional regulation from the other regulator genes, for example *flhF*.

Flagellar assembly uses a secretion apparatus similar to type III secretion systems. This is dependent upon export chaperones that protect and transport structural subunits using the membrane-associated export ATPase, FliI [38,39]. Therefore, the decreased transcription of *fliI *might be another factor in blocking motility via shortened filament length in the Δ*luxS*_Hp _mutant as *Helicobacter fliI *mutants are non-motile and synthesise reduced amounts of flagellin (FlaA, FlaB) and hook protein (FlgE) subunits [38].

In our experiments, the motility defect, down-regulated flagellar gene expression and reduced synthesis of flagellar proteins in the Δ*luxS*_Hp _mutant were due to loss of AI-2 only, and not to the metabolic effect of *luxS*_Hp _on biosynthesis of cysteine. These results suggest that LuxS/AI-2 is likely to be a functional signalling system contributing to control motility in *H. pylori*. However, it is still uncertain whether AI-2 functions as a true QS signal in *H. pylori*, in part because there are no genes encoding proteins that can be confidently identified as components of an AI-2 sensory and regulatory apparatus in *H. pylori *[13,40]. Also, we cannot exclude the possibility that AI-2 acts through other undefined effects and not as a signalling molecule, although as it is known to have similar effects through signalling in other bacteria, this appears unlikely.

*Campylobacter jejuni *also possesses a *luxS *homologue and produces AI-2. Inactivation of *luxS *in a *C. jejuni *strain (81-176) also resulted in reduced motility and affected transcription of some genes [41]. However, despite its effect on signalling, AI-2 does not function as a QS molecule in *C. jejuni *(NCTC 11168) during exponential growth *in vitro *when a high level of AI-2 is produced [42]. Thus, so far there is no good evidence to ascertain whether AI-2 functions as a true QS signal in this species. In *H. pylori*, Lee *et al. *and Osaki *et al. *looked at fitness of Δ*luxS*_Hp _mutants *in vivo *using mouse and gerbil models, respectively [18,19]. The authors did not favour a QS or even a signalling explanation for the reduced fitness mechanisms but both speculated that it might be caused by metabolic disturbances upon loss of *luxS*_Hp _[18,19]. However, it could potentially be explained by reduced signalling leading to reduced motility, and given the ecological niche of *H. pylori *there would be logic to a signalling (perhaps even QS) system increasing motility. For example, we speculate that if a microcolony of *H. pylori *in a particular area of the stomach reached a critical size it would be potentially advantageous for flagellar biogenesis to be enhanced so that highly motile bacteria could disseminate to new regions of the stomach. If this hypothesis was confirmed, it would have important implications for *H. pylori *virulence and for the spread of infection within and between people.

## Conclusions

Our study suggests that as well as being a metabolic enzyme in the reverse transsulphuration pathway, *H. pylori *LuxS has a second role in regulation of motility by modulating flagellar transcripts and flagellar biosynthesis. This is achieved through production of the signalling molecule AI-2, rather than the metabolic effect of LuxS in cysteine biosynthesis.

## List of abbreviations

AMC: activated methyl cycle; AI-2: auto-inducer-2; CFS: cell free supernatant; DPD: 4, 5-dihydroxy-2, 3-pentanedione; QS: quorum sensing; RD: reflective density; RTSP: reverse transsulphuration pathway.

## Authors' contributions

JCA and KRH contributed to the design and supervision of the study. FS participated in the design of experiments, carried out the study, analysed data and drafted the manuscript. LH and RES contributed to the work of microscopy and flagellar morphology, and wrote the related section of the manuscript. ND contributed to the construction of the Δ*luxS *mutant. JTL and TLC designed and generated the plasmids needed for the construction of the complemented Δ*luxS*^+ ^mutant. KRH, RES, TLC, LH and ND gave useful comments to the manuscript. JCA and FS coordinated the manuscript to the final version. All authors read and approved the final manuscript.
